# Alterations in hematologic indices during long-duration spaceflight

**DOI:** 10.1186/s12878-017-0083-y

**Published:** 2017-09-08

**Authors:** Hawley Kunz, Heather Quiriarte, Richard J. Simpson, Robert Ploutz-Snyder, Kathleen McMonigal, Clarence Sams, Brian Crucian

**Affiliations:** 1 KBRwyle, 2400 NASA Parkway, Houston, TX 77058 USA; 2 Louisiana State University, Baton Rouge, Louisiana, 70803 USA; 3 University of Houston, 4800 Calhoun Rd, Houston, TX 77004 USA; 4 University of Michigan School of Nursing, 400 North Ingalls Building, Ann Arbor, MI 48109 USA; 5 NASA Johnson Space Center, 2101 E NASA Parkway, Houston, TX 77058 USA

**Keywords:** Spaceflight, Red blood cells, Anemia, Platelets

## Abstract

**Background:**

Although a state of anemia is perceived to be associated with spaceflight, to date a peripheral blood hematologic assessment of red blood cell (RBC) indices has not been performed during long-duration space missions.

**Methods:**

This investigation collected whole blood samples from astronauts participating in up to 6-months orbital spaceflight, and returned those samples (ambient storage) to Earth for analysis. As samples were always collected near undock of a returning vehicle, the delay from collection to analysis never exceeded 48 h. As a subset of a larger immunologic investigation, a complete blood count was performed. A parallel stability study of the effect of a 48 h delay on these parameters assisted interpretation of the in-flight data.

**Results:**

We report that the RBC and hemoglobin were significantly elevated during flight, both parameters deemed stable through the delay of sample return. Although the stability data showed hematocrit to be mildly elevated at +48 h, there was an in-flight increase in hematocrit that was ~3-fold higher in magnitude than the anticipated increase due to the delay in processing.

**Conclusions:**

While susceptible to the possible influence of dehydration or plasma volume alterations, these results suggest astronauts do not develop persistent anemia during spaceflight.

## Background

A number of physiologic changes are known to occur during prolonged spaceflight. The combined effects of microgravity, radiation, physical and psychological stressors, altered nutrition, disrupted circadian rhythms, and other factors have impacts on many of the body’s systems, including vision, the musculoskeletal system, and the immune system [1]. Another marked alteration in physiology is the redistribution of fluids upon entering microgravity, which in turn may influence various hematologic parameters.

Without constant gravitational force, an almost immediate shift of fluids toward the head occurs, resulting in a “puffy” face and a reduced leg volume. An “acute plethora” of blood surrounds the central organs as peripheral blood is no longer held in the extremities by gravity [2–4]. While there is relevant existing information regarding red blood cells and spaceflight, it is primarily associated with short-duration Space Shuttle missions. Hematocrit, red blood cell (RBC) count, hemoglobin and plasma volume have been measured during short-duration spaceflight. RBC count and hemoglobin were found to be elevated throughout a 14-day mission, while plasma volume was found to be decreased 17% within the first 24 h immediately after launch, and remained depressed when measured at flight day 8 [2, 4]. In the same subjects, RBC mass was measured, but only immediately after landing, at which time a reduction in RBC mass was found [2–4]. The authors ascribed the likely cause of the reduction in RBC mass to be the “acute plethora” of RBCs resulting from fluid shifts during flight. These reductions in RBC mass following spaceflight have been observed throughout the history of spaceflight [3, 5]. During 10 to 14-day space missions, average losses of 10% to 15% of RBC mass immediately on landing are consistently reported, corresponding to a loss of approximately 1% RBC mass per day [3, 5]. These summary alterations result in an approximate 10% decrease in total blood volume [1] following short duration flight. Similar reductions have been observed in post-flight samples obtained after long-duration spaceflight [3, 5–7]. A reduction in RBC mass during spaceflight, termed “spaceflight anemia,” is therefore a generally accepted phenomenon and appears to be a normal adaptation to microgravity [3, 5].

A majority of the studies examining alterations in RBC mass have been limited to post-flight evaluations. The few in-flight evaluations have been limited to short-duration flights, throughout which physiologic adaptations to microgravity are likely to still be occurring. Findings during short-duration flight may therefore not accurately reflect the in-flight condition during long-duration flight. As hematology indices generally do not tolerate freezing and ambient blood samples are rarely returned from space, there is a dearth of in-flight hematologic indices during long-duration spaceflight. The evidence that does exist for long-duration spaceflight appears to indicate that the reductions in RBC mass may actually be less severe for longer missions [7]. Further, very little information regarding the effects of spaceflight on platelets is available [3]. Therefore, additional data describing the in-flight hematologic condition as the body adapts to long-duration spaceflight are needed.

Here we report RBC and platelet indices on blood collected before, *during*, and after long-duration spaceflight as a subset of a two parent investigations of the effects of long-duration spaceflight on the immune system [8]. In-flight samples were collected in conjunction with crew returns and returned to the laboratory within 48 h, enabling an examination of ambient blood samples collected on board the International Space Station (ISS). A standard complete blood count (CBC) was performed on all samples. Alterations in the bulk leukocyte subsets during spaceflight, including in- and post-flight elevations of white blood cell and granulocyte concentrations, were previously reported alongside additional white blood cell functional data [8]. Here, in-flight hematologic indices were examined in an effort to better understand in-flight alterations in RBC and platelet parameters during long-duration spaceflight. To accurately interpret the data and to determine the impact of the processing delay resulting from the time required to transport ambient blood from the International Space Station (ISS) to the laboratory, a stability study examining the effects of room-temperature blood storage on these indices was also performed.

## Methods

### Subjects

Thirty-one astronaut crewmembers (25 males, 6 females, mean age 52 years, range 38–61) participated in one of two parent investigations, the National Aeronautics and Space Administration (NASA) ‘Integrated Immune’ and the University of Houston ‘Salivary Markers’ studies onboard the ISS. Of the 31 crewmembers, 24 flew on the Russian Soyuz capsule and completed missions of approximately 6 months. The remaining 7 crewmembers rotated to the ISS via the United States Space Shuttle. Of those 7, 5 completed missions lasting greater than 100 days, and 2 had mission durations of less than 60 days.

To determine the effects of room temperature storage on hematologic indices, 20 healthy, adult, non-astronaut subjects (12 males, 8 females, mean age 45 ± 13 years, range 26–65) were recruited for a stability study by the NASA Johnson Space Center (JSC) Test Subject Facility. For all astronaut and stability study subjects, approval was obtained from the JSC Institutional Review Board and written informed consent was obtained from all subjects.

### Blood sampling

For both the flight study and the stability study, peripheral blood was collected into a 10.0 mL ethylenediaminetetraacetic acid (EDTA) spray-coated blood collection tube (BD, Franklin Lakes, NJ, USA). Pre-flight samples were collected at approximately 180 days (L-180) and 45 days (L-45) prior to launch. In-flight, samples were collected within the first 2 weeks of flight (early), between months 2 and 4 of the mission (mid), and approximately 6 months into the mission, immediately prior to return (late). For those astronauts completing shorter duration missions, only 2 samples were collected and corresponded to the “early” and “mid” time points. Post-flight, samples were collected within 3–8 h post-landing (R + 0) and 30 days post-flight (R + 30). Stability subject samples consisted of a single 10.0 mL EDTA spray-coated blood collection tube (BD), sampled as indicated following.

### Processing

**Fig. 1:**
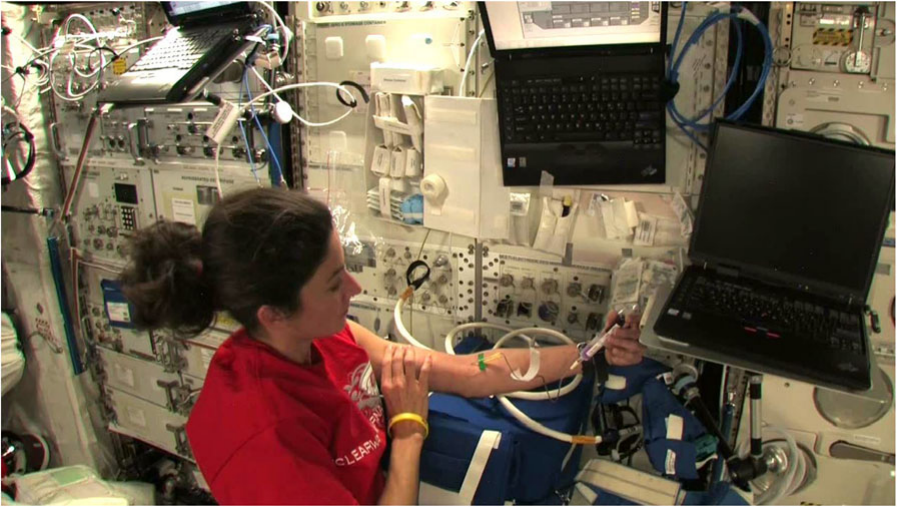
Blood collection onboard the ISS. Astronaut Nicole Stott performs phlebotomy on the ISS. Samples were collected ~10 h prior to return vehicle undocking (Space Shuttle or Soyuz). Blood samples were returned to the laboratory for analysis within 48 h of collection

All CBCs were performed using calibrated, automated hematology analyzers (JSC processing: Coulter LH750, Miami, FL, USA; Kennedy Space Center (KSC) processing: Coulter Gen-S, Miami, FL, USA; Star City, Russia processing: ABX Pentra, Horiba Medical, Irvine, CA, USA; University of Houston Processing: Mindray BC3200, Mindray, Shenzhen, China). Upon arrival to the laboratory, a 1.0 mL aliquot was removed for CBC analysis. All pre- and post-flight astronaut blood samples were immediately processed at JSC; however, the analysis of samples collected in-flight was delayed up to 48 h as a result of the time required to transport the ambient blood from the ISS to the laboratory. Briefly, blood samples were collected from each participating crewmember onboard the ISS (Fig. 1) approximately 10 h prior to hatch closure of the returning vehicle (either Shuttle or Soyuz). Collected blood samples were stored in customized blood pouches and transferred to the returning vehicle for return to Earth. Processing of in-flight samples was performed at JSC or the University of Houston, KSC, or at Star city, Russia, depending on the mission landing site.

To examine the effects of the processing delay on the in-flight samples, sterile syringes were used to obtain 1.0 mL aliquots from the 10.0 mL EDTA-coated blood collection tubes collected from healthy donors. The first CBC was run immediately following blood collection from each stability study subject. Subsequently, blood was stored in the dark at room temperature, and 1.0 mL aliquots were removed and analyzed at 24, 48, and 72 h post-collection. All stability samples were processed and analyzed at JSC.

### Statistical analysis

This was a longitudinal, repeated-measures study examining the effects of spaceflight on multiple hematologic parameters. Each astronaut served as his/her own control and all in-flight and post-flight time points were compared to the astronaut’s baseline sample. The L-180 time point was considered baseline, as pre-mission stressors may have influenced the L-45 time point. The distribution of each parameter was tested for normality using the Shapiro-Wilk Normality Test. Non-normal data were transformed logarithmically and outliers were removed for analysis. For all RBC indices, mixed-effects linear models were used to compare each subsequent time point to the L-180 baseline. A random intercept was used to account for the repeated-measures design of the study. Statistical analysis was performed using STATA statistical software (v14, StataCorp LP, College Station, TX, USA). Significance was set at *p* < 0.05.

To determine the stability of the hematologic indices, two one-sided tests of equivalency for dependent samples were performed on the data from the 20 healthy stability study subjects, comparing each of the aged samples to the corresponding Day 0 baseline sample. The within-person coefficient of variation for each hematologic parameter reported by Lacher et al. [9] was used to define the equivalence bounds for the two one-sided tests. Significant results (*p* < 0.05) in the two one-sided tests indicate that the aged samples and the baseline sample are practically equivalent. Results from the stability study were used to inform results from the astronaut study and assist with interpretation, but no direct comparisons were made between the astronauts and the stability study subjects. The stability study statistical calculations were performed using Microsoft Excel and the spreadsheet developed by Lakens [10].

## Results

**Fig. 2:**
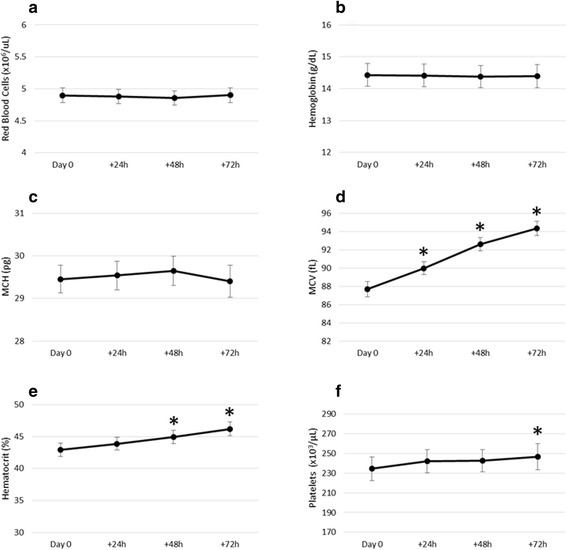
Hematologic indices evaluated immediately following blood collection, and 24, 48, and 72 h after collection. All aged samples were compared to the baseline sample analyzed immediately post-collection using two one-sided tests for dependent samples. Data are presented as mean ± standard error. Samples that were not statistically considered equivalent to the baseline sample (*p* > 0.05) are indicated with *. **a** Red blood cell concentration (×10^6^ cells/μL); **b** hemoglobin concentration (g/dL); **c** mean corpuscular hemoglobin (MCH; pg); **d** mean corpuscular volume (MCV; fL); **e** hematocrit (%); and **f** platelet concentration (×10^3^ cells/μL). All parameters were measured using calibrated automated hematology analyzers

Of the RBC and platelet indices included in a CBC, the hematology analyzers measure RBC count, mean corpuscular volume (MCV), hemoglobin, and platelet concentration. All other parameters are calculated from these measurements. Only the RBC count, hemoglobin, mean corpuscular hemoglobin (MCH), and platelet concentration remained stable for 48 h at room temperature (Fig. 2a-c, f). Parameters were considered stable if, when compared to the baseline sample, they were significantly within the pre-defined equivalent bounds (*p* < 0.05) at the 24 and 48 h time points. At both 24 and 48 h after collection, when compared to the baseline sample, the platelet concentration fell within the pre-defined equivalent bounds (*p* < 0.05); however, at 72 h after collection, the platelet concentration was no longer significantly practically equivalent to the baseline sample (t(19) = −1.554, *p* = 0.068). Both hematocrit and MCV steadily increased over the 72 h of storage at room temperature (Fig. 2d and e). Compared to baseline, MCV was not within the equivalent bounds at 24 h (t(19) = 6.337, *p* = 1.000). While elevated at 24 h, hematocrit was significantly within the equivalent bounds (t(19) = −1.885, *p* = 0.037); however, hematocrit was not significantly within the equivalent bounds by 48 h (t(19) = 0.75, *p* = 0. 076). Given the relationship between hematocrit, MCV, and RBC count (hematocrit = [MCV × RBC count]/10) alterations in MCV will necessarily affect the hematocrit values. The elevations in hematocrit over the 72 h therefore reflect the elevations in MCV. Additional parameters that were measured but not included in any subsequent analysis due to instability following delayed processing include red cell distribution width, mean corpuscular hemoglobin concentration, and mean platelet volume (data not shown).

**Fig. 3:**
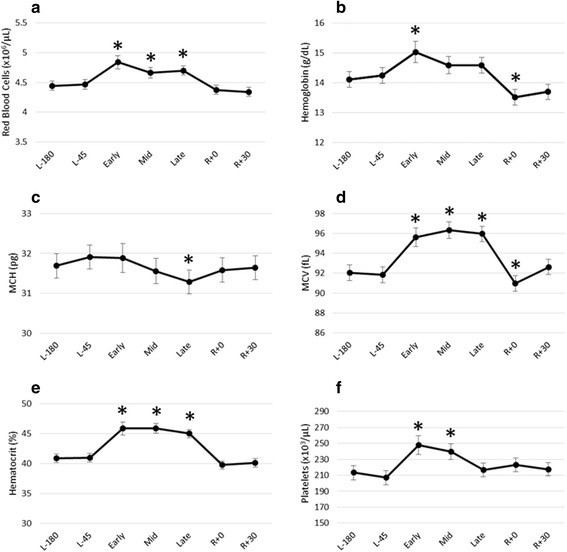
Hematologic indices evaluated before, during, and after spaceflight. All samples were compared to the L-180 baseline time point using a linear mixed model with random intercept. Data are presented as mean ± standard error. Significant differences from the L-180 baseline (*p* < 0.05) are indicated with *. **a** Red blood cell concentration (×10^6^ cells/μL); **b** hemoglobin concentration (g/dL); **c** mean corpuscular hemoglobin (MCH; pg); **d** mean corpuscular volume (MCV; fL); **e** hematocrit (%); and **f** platelet concentration (×10^3^ cells/μL). All parameters were measured using calibrated automated hematology analyzers

All astronaut samples drawn on the ISS were returned to the lab within 48 h, and the majority of samples were returned by ~37 h post-collection. Therefore, only parameters that remained stable at 48 h were included in the analysis of the effects of long-duration spaceflight on hematologic indices, with the exception of hematocrit and MCV, discussed below. The effects of long-duration spaceflight on the analyzed hematologic indices are presented in Fig. 3a-f. All parameters remained consistent prior to flight, with no significant differences between the L-180 and L-60 time points. RBC concentration was significantly elevated at all three in-flight time points compared to the L-180 baseline time point (Fig. 3a; L-180: mean 4.4 ± 0.4, range 3.5–5.1; Early: mean 4.8 ± 0.5, range 3.9–5.7; Mid: 4.7 ± 0.4, range 3.9–5.4; late: 4.7 ± 0.4, range 4.1–5.6). Hemoglobin was elevated early in flight compared to L-180, but returned to pre-flight values as the mission progressed (Fig. 3b; L-180: mean 14.1 ± 1.4, range 11.0–17.8; Early: mean 15.0 ± 1.9, range 10.7–17.5). Throughout the mission, MCH decreased, and was significantly lower than the L-180 baseline by the late-flight time point (Fig. 3c; L-180: mean 31.7 ± 1.6, range 28.8–36.4; Late: 31.3 ± 1.9, range 26.3–34.0). While hemoglobin fell below L-180 baseline values on landing day (Fig. 3b; L-180: mean 14.1 ± 1.4, range 11.0–17.8; R + 0: mean 13.5 ± 1.4, range 10.1–15.9), RBC count and MCH returned to pre-flight values upon re-entry, and by R + 30, all indices were at pre-flight levels.

Significant increases in MCV observed in flight (3.9%, 4.6%, and 4.2% increases in mean values compared to the L-180 baseline at early, mid, and late, respectively; Fig. 3d), reflect the changes observed following the 48 h processing delay (5.6% increase in mean values from baseline to +48 h; Fig. 2d). Therefore, we do not identify any MCV variations attributable to spaceflight. As noted previously, elevations in MCV will also manifest as elevations in hematocrit. Although hematocrit values increased when subjected to processing delays and were significantly elevated at 48 h after collection (Fig. 2e), the alterations in hematocrit during spaceflight were striking (Fig. 3e). The significant (*p* < 0.05) elevations in hematocrit observed in flight were of greater magnitude than those observed simply from the elevations in MCV as a result of the processing delay. A 4.7% increase in the mean hematocrit was observed after the 48 h processing delay (Day 0), while the percentage increases in mean hematocrit at the early, mid, and late time points compared to the L-180 time point were 12.2%, 12.2%, and 10.0%, respectively (L-180 mean 40.9 ± 3.9, range 33.1–48.0; Early: mean 45.9 ± 4.7, range 38.2–52.1; Mid: 45.9 ± 5.5, range 38.9–58.3; Late: 45.0 ± 2.5, range 38.9–49.9). These in-flight elevations are therefore most likely due to a combination of a true in-flight increase in RBC count and an artifactual increase in MCV resulting from the processing delay.

Platelet concentration was elevated early in flight. While tracking toward recovery, platelet concentration remained significantly elevated at the mid-flight time point, but was not significantly higher than pre-flight by the late time point (Fig. 3f). The concentration remained stable upon landing and during recovery.

## Discussion

While spaceflight anemia has been consistently reported post-flight and during short-duration flight [3, 5], little is known about the in-flight condition during long-duration missions. In this study, we observed statistically significant elevations in the concentrations of RBCs, platelets, and hemoglobin, and we interpret an apparent increase in hematocrit at multiple time points during long-duration spaceflight.

The alterations associated with spaceflight observed in this study are in accordance with previous findings of elevated RBC indices in-flight. RBC concentration, hemoglobin, and hematocrit have been shown to be elevated during the first few days of flight [2, 4, 11]; however, here we show that RBC concentration remains elevated even after the initial period of adaptation to microgravity. Although previous findings suggest that RBC mass is decreased in association with spaceflight [2–4, 7], alterations in cell mass and concentration need not track together. While the observed elevations in RBC concentration and hematocrit may simply be due to greater losses in plasma volume than in RBC mass, it is possible that RBC mass is partially restored as the body adjusts to the absence of gravity as flight duration extends, and the losses in RBC mass are less severe during long-duration spaceflight. In a review of literature on RBC mass and spaceflight, Tavassoli et al. [3] noted that in the first 3 weeks of flight, length of flight and losses in RBC mass were positively correlated, with greater losses in RBC mass occurring in longer flights; however, in the studies performed on the longer-duration Skylab 2, 3 and 4 missions (28, 59, and 84 days, respectively), the longer missions were actually associated with smaller decreases in RBC mass [3, 7]. Therefore it has been previously postulated that during prolonged exposure to microgravity a new RBC mass homeostasis is reached, and the early reductions in RBC mass are abrogated [5, 12].

The observed reduction in MCH late in-flight may be reflected in the relationship between RBC concentration and hemoglobin, as RBC concentration remained elevated throughout the flight while hemoglobin was significantly elevated only early in the flight. A reduced requirement for oxygen-carrying capabilities and easier delivery of oxygen to tissues while in microgravity may drive some of these changes [5, 6].

Previous post-flight findings are varied, as both elevations [7, 13] and depressions [7, 11] in RBC count, hemoglobin, and hematocrit have been reported. Here we found significant post-flight decreases in hematocrit and MCV, while all other parameters rapidly returned to baseline upon re-entry. Interestingly, immediately after the 28-day Skylab 2 mission RBC count, hemoglobin concentration and hematocrit fell below pre-flight values, and while RBC count had recovered by day 7 post-flight, hematocrit and hemoglobin concentration were still below pre-flight levels at 18 days post-flight [7]. In contrast, on the Skylab 3 and 4 missions (59 and 84 days, respectively) RBC count, hemoglobin concentration, and hematocrit were elevated immediately upon landing, but subsequently began to decline and were significantly lower than pre-flight values 3 days after landing, returning to normal in the 3 week testing period following the flights [7]. With the dependence of these indices on plasma volume, the timing of the sample and the conditions of the return may have a large impact. Both dehydration and plasma volume shifts upon re-entry into gravity can significantly affect these parameters. Plasma volume has been shown to be rapidly restored upon re-entry [14, 15], which may account for the rapid return to baseline values of RBC count observed in this study, given the in-flight elevations in these parameters; however, without an accurate measure of plasma volume, it is difficult to make any conclusive statements. Additional sampling between the R + 0 and the R + 30 samples may be beneficial in determining the erythrokinetics post-flight. Depressions in RBC count, hemoglobin concentration, and hematocrit in the weeks after spaceflight were reported after the Skylab missions and by others [2, 7, 11, 14] and were interpreted as potential depressions in red blood cell mass during spaceflight that were slower to recover upon return to Earth than the depressions in plasma volume. Monitoring the RBC indices in the days following flight in the current study would have provided interesting information, given the observed in-flight elevations, and not depressions, in various hematologic indices.

Little data exist regarding in-flight platelet concentrations [3]; however, the reports that do exist suggest that microgravity and simulated microgravity actually induce a state of thrombocytopenia [16, 17]. In contrast, the elevations in platelet concentration observed in this investigation at the early and mid-flight time points may be due to reductions in plasma volume without any true increase in platelet numbers. The gradual return toward baseline of platelet concentration over the course of the 6-month mission may be indicative of a homeostatic mechanism that serves to counteract elevations in platelet concentration resulting from reduced plasma volume. Interestingly, BE Crucian, SR Zwart, S Mehta, P Uchakin, HD Quiriarte, D Pierson, CF Sams and SM Smith [18] recently reported that plasma thrombopoietin, which stimulates platelet production and is generally elevated when platelet levels are low, was elevated throughout 6-months of orbital spaceflight; however, vascular endothelial growth factor (VEGF) and C-X-C motif chemokine 5 (CXCL5), both of which are platelet-derived and positively correlated with platelet concentration [19, 20], were also elevated throughout the 6-month missions [18]. The elevations in plasma VEGF and CXCL5 [16], in conjunction with the finding that platelet concentration was also elevated, appears to indicate that long-duration spaceflight does not induce thrombocytopenia; however, the discrepant finding that thrombopoietin was also elevated [16] warrants further investigation.

Although the performance of a CBC on samples collected during spaceflight generated novel information, these findings must be interpreted with caution. The cellular concentrations are dependent on plasma volume, and therefore the observed elevations may be influenced by reductions in plasma volume without any real increase in cellular mass. Indeed, plasma volume has been shown to decrease by approximately 17% within the first 24 h of spaceflight [2]; however, like changes in RBC mass, the alterations in plasma volume have been primarily observed during short-duration flight or post-flight, and little evidence exists describing changes in plasma volume during long-duration spaceflight. The reductions in plasma volume observed between flight days 8 and 12 by Alfrey et al. [2], while still significant, were smaller than the reductions observed on the first flight day, indicating there may be a continued trend toward plasma volume recovery as time on board the ISS progresses. In a comparison of short and long-duration flights, the average loss in plasma volume for 5 long-duration astronauts was marginally lower than the average loss in 29 short-duration astronauts, though this was not statistically significant [21]. To fully interpret the alterations presented in the current study, plasma volume must also be assessed during long-duration space flight.

The measurement of erythropoietin (EPO) in-flight would also aid in the interpretation of the reported findings; unfortunately EPO was not determined as part of the parent immune investigations. EPO controls RBC mass by regulating the rate of division of RBC progenitors in the bone marrow, and it has also been postulated to play a role in the neocytolysis process by which newly released RBCs are selectively destroyed upon entering into microgravity [12, 15, 22]. EPO has been shown to be reduced early in-flight but elevated following short-duration flight [4], indicating that homeostatic mechanisms attempt to reduce RBC mass upon entering microgravity and restore it upon landing. However, to our knowledge, EPO has not been measured during long-duration flight. The measurement of EPO in future studies of prolonged spaceflight may help to explain the present findings of elevated RBC count throughout long-duration flight.

The delay in processing for the in-flight blood samples is also a limitation of the study. RBC, hemoglobin and platelet concentration have all been shown to be stable for up to 72 h when blood samples collected with EDTA are stored at 4 °C [23]; however, blood samples for our investigations were returned at ambient temperature. Despite the recommendations that samples be refrigerated, results of the stability tests indicate that RBC count, hemoglobin concentration, MCH values, and platelets remain stable for at least 48 h, even at room temperature. The elevations in hematocrit and MCV reported here are in accordance with other study findings. MCV begins to increase within 6–12 h of blood collection, which, in turn, causes an elevation in hematocrit without any alterations in RBC concentration or plasma volume, even in refrigerated samples [23]. While the elevations in hematocrit and MCV hinder our analysis of the in-flight data, the stability of RBC count, hemoglobin, MCH, and platelet concentration over 48 h indicates that the observed alterations in these parameters are likely caused by factors associated with space-flight, and are not the result of delayed sample processing.

## Conclusions

Spaceflight anemia is a widely reported phenomenon; however, the vast majority of evidence demonstrating reductions in RBC mass has been collected post-flight. To our knowledge, this is one of the first studies to examine hematologic parameters on blood samples collected during long-duration spaceflight. The data suggest that spaceflight anemia may be less of a concern during long-duration spaceflight. However, as previously noted, the fluctuations in these concentration-dependent variables are influenced by changes in plasma volume. Despite this limitation, the sustained elevation of RBC and platelet concentrations throughout a 6-month mission on board the ISS reported here seems to warrant further investigation, and accurate in-flight assessments of plasma volume during long-duration spaceflight would aid in the interpretation of the findings of this study.

## Abbreviations

CBC: Complete blood count
CXCL5: C-X-C motif chemokine 5
EDTA: Ethylenediaminetetraacetic acid
EPO: Erythropoietin
ISS: International Space Station
JSC: Johnson Space Center
KSC: Kennedy Space Center
MCH: Mean corpuscular hemoglobin
MCV: Mean corpuscular volume
NASA: National Aeronautics and Space Administration
RBC: Red blood cell
VEGF: Vascular endothelial growth factor
